# Glycyrrhizin Effectively Inhibits SARS-CoV-2 Replication by Inhibiting the Viral Main Protease

**DOI:** 10.3390/v13040609

**Published:** 2021-04-02

**Authors:** Lukas van de Sand, Maren Bormann, Mira Alt, Leonie Schipper, Christiane Silke Heilingloh, Eike Steinmann, Daniel Todt, Ulf Dittmer, Carina Elsner, Oliver Witzke, Adalbert Krawczyk

**Affiliations:** 1 Department of Infectious Diseases, West German Centre of Infectious Diseases, Universitätsmedizin Essen, University Duisburg-Essen, 45147 Essen, Germany; lukas.vandesand@uk-essen.de (L.v.d.S.); Maren.Bormann@uk-essen.de (M.B.); Mira.Alt@uk-essen.de (M.A.); Leonie.Schipper@uk-essen.de (L.S.); Christiane.Heilingloh@uk-essen.de (C.S.H.); Oliver.Witzke@uk-essen.de (O.W.); 2 Department of Molecular and Medical Virology, Faculty of Medicine, Ruhr University Bochum, 44801 Bochum, Germany; Eike.Steinmann@ruhr-uni-bochum.de (E.S.); Daniel.todt@ruhr-uni-bochum.de (D.T.); 3 Institute for Virology, University Hospital Essen, University Duisburg-Essen, 45147 Essen, Germany; Ulf.Dittmer@uk-essen.de (U.D.); carina.elsner@uk-essen.de (C.E.)

**Keywords:** SARS-CoV-2, glycyrrhizin, main protease

## Abstract

The outbreak of SARS-CoV-2 developed into a global pandemic affecting millions of people worldwide. Despite one year of intensive research, the current treatment options for SARS-CoV-2 infected people are still limited. Clearly, novel antiviral compounds for the treatment of SARS-CoV-2 infected patients are still urgently needed. Complementary medicine is used along with standard medical treatment and accessible to a vast majority of people worldwide. Natural products with antiviral activity may contribute to improve the overall condition of SARS-CoV-2 infected individuals. In the present study, we investigated the antiviral activity of glycyrrhizin, the primary active ingredient of the licorice root, against SARS-CoV-2. We demonstrated that glycyrrhizin potently inhibits SARS-CoV-2 replication in vitro. Furthermore, we uncovered the underlying mechanism and showed that glycyrrhizin blocks the viral replication by inhibiting the viral main protease M^pro^ that is essential for viral replication. Our data indicate that the consumption of glycyrrhizin-containing products such as licorice root tea of black licorice may be of great benefit for SARS-CoV-2 infected people. Furthermore, glycyrrhizin is a good candidate for further investigation for clinical use to treat COVID-19 patients.

## 1. Introduction

The newly emerged coronavirus, which was designated as severe acute respiratory syndrome coronavirus 2 (SARS-CoV-2), is the causative agent of the COVID-19 disease. Even presymptomatic patients or patients with mild symptoms are able to infect other people. The most common symptoms are headache, fever, cough, loss of taste and smell, sore throat and rhinorrhea. Highly effective and well-tolerated medication for hospitalized and non-hospitalized patients is urgently needed. Besides compounds that were initially approved for the treatment of other viral infections such as remdesivir [1], traditional herbal medicine substances were discussed as promising candidates for the complementary treatment of viral diseases and recently suggested for the treatment of COVID-19. Lastly, an in-silico simulation study proposed an antiviral activity of glycyrrhizin against SARS-CoV-2, but this hypothesis remains experimentally unproven up to now [2]. Glycyrrhizic acid is a triterpene saponin and found in high concentrations in the root of the Glycyrrhiza glabra plant. It was described as an antiviral active ingredient of the licorice root and exhibits antiviral activity against herpes simplex viruses [3], the human immunodeficiency virus as well as human and animal coronaviruses [4]. In the present study, we investigated aqueous licorice root extract for its antiviral activity against SARS-CoV-2 in vitro, identified the active compound glycyrrhizin and uncovered the respective mechanism of how glycyrrhizin inhibits viral replication. We demonstrated that glycyrrhizin, the primary active ingredient of the licorice root, potently inhibits SARS-CoV-2 replication in vitro. Subsequently, we examined the underlying mechanism of antiviral efficacy and demonstrated that glycyrrhizin blocks the viral replication by inhibiting the viral main protease. Our experiments highlight glycyrrhizin as a potential antiviral compound that should be further investigated for the treatment of COVID-19.

## 2. Materials and Methods

### 2.1. Licorice Root Extract

Dried licorice roots (Teeversand Naturideen, Hennstedt, Germany) were brewed in PBS at a concentration of 8 mg/mL (*w/v*). The fluid was subsequently sterile filtered with a syringe filter (Minisart® NML Plus 0.2 μm, Sartorius, Goettingen, Germany) to obtain an aqueous licorice root extract.

### 2.2. Glycyrrhizin Acid Ammonium-Nitrate

For cell culture experiments, glycyrrhizin acid ammonium-nitrate (Roth, Karlsruhe, Germany) was dissolved in DMEM containing 2% (*v*/*v*) FCS, penicillin (100 IU/mL) and streptomycin (100 µg/mL) at 37 °C and adjusted with NaOH to pH 7. Otherwise, glycyrrhizin was dissolved in water and stored at 37 °C.

### 2.3. Cells and Virus

Vero E6 cells (American Type Culture Collection, ATCC, Manassas, Virginia, USA; ATCC® CRL-1586™) were maintained in Dulbecco’s modified Eagle’s medium (DMEM Life Technologies Gibco, Darmstadt, Germany), supplemented with 10% fetal calf serum (FCS; Life Technologies Gibco, Darmstadt, Germany), penicillin (100 U/mL) and streptomycin (100 µg/mL) at 5% CO_2_ and 37 °C. The clinical SARS-CoV-2 isolate was obtained from a nasopharyngeal swab of a patient suffering from COVID-19 disease at our hospital. The virus was propagated in Vero E6 cells cultured in DMEM supplemented with 10% (*v*/*v*) FCS, penicillin (100 IU/mL), streptomycin (100 μg/mL), ciprofloxacin (10 µg/mL) and amphotericin B (2.5 µg/mL) and stored at −80 °C. Viral titers were determined using a standard endpoint dilution assay and calculated as 50% tissue culture infective dose (TCID_50_)/mL, as previously described [5].

### 2.4. Antiviral Activity of Aqueous Licorice Root Extract and Glycyrrhizin

The antiviral activity of aqueous licorice root extract and glycyrrhizin was determined in cell culture using endpoint dilution. For this purpose, serial dilutions of licorice root extract or glycyrrhizin (0.004–4 mg/mL) were pre-incubated with 100 TCID_50_ of SARS-CoV-2 for 1 h at 37 °C and subsequently incubated on confluent Vero E6 cells grown in 96-well microtiter plates (combined pre- and post-entry approach). After 48 h, the cells were stained with crystal violet (Roth, Karlsruhe, Germany) solved in 20% methanol (Merck, Darmstadt, Germany) and analyzed for cytopathic effects (CPE). The neutralizing titer was determined as the concentration required for reducing virus-induced CPE by 100%.

Moreover, the antiviral activity of glycyrrhizin was investigated under post-entry conditions (post-entry treatment). Therefore, Vero E6 cells were infected with 100 TCID_50_ SARS-CoV-2 for 4 h and subsequently treated with various glycyrrhizin concentrations ranging from 0.002–4 mg/mL (post-entry conditions). After 2 days of incubation, the cells were stained with crystal violet (Roth, Karlsruhe, Germany) solved in 20% methanol (Merck, Darmstadt, Germany) and the CPEs were scored using light microscopy.

### 2.5. Cell Viability Assay

Cytotoxicity of licorice root extract and glycyrrhizin towards Vero E6 cells was determined by using the “Orangu cell counting solution” (Cell guidance systems, Cambridge, UK) according to the manufacturer’s protocol. Orangu™ is a colorimetric assay for the determination of viable cell numbers in cytotoxicity assays. Orangu™ utilizes WST-8, a water-soluble tetrazolium salt that is reduced by dehydrogenase activities in viable cells. The amount of the orange-colored formazan dye formed is directly proportional to the number of living cells [6]. Briefly, descending licorice root extract or glycyrrhizin concentrations (0.002–4 mg/mL) were incubated (37 °C, 5% CO_2_) with 1 × 10^5^ Vero E6 cells per ml (and 10,000 cells/well of a 96-well plate) grown in 96-well microtiter plates. At four distinct time points (5 min, 12 h, 24 h and 4 h), medium (DMEM containing 2% (*v*/*v*) FCS (PAA), penicillin (100 IU/mL) and streptomycin (100 µg/mL)) was changed and 10 µL of Orangu™ cell counting solution was added for 120 min of incubation (37 °C, 5% CO_2_). Cell viability was measured at an absorbance of 450 nm using Mithras LB 940 (Berthold Technologies, Oak Ridge, TN, USA) and normalized to untreated control cells.

### 2.6. Determination of EC_50_ of Glycyrrhizin

To further investigate the antiviral efficacy of glycyrrhizin, we determined the half-maximal effective concentration (EC_50_) sufficient to inhibit viral replication by 50%. Confluent Vero E6 cells grown in 6-well plates were infected with 1 × 10^4^ TCID_50_ SARS-CoV-2 and treated with various concentrations of glycyrrhizin ranging from 0.0625 to 4 mg/mL. After 48 h of incubation, the supernatants were harvested and the viral loads were determined using endpoint dilution. The experiment was performed in triplicates and EC_50_ was calculated via linear regression using GraphPad Prism 8.0.1. (Graph Pad Software, San Diego, CA, USA).

### 2.7. Quantitative SARS-CoV-2 RT-PCR

Confluent Vero E6 cells grown in 24-well plates were infected with 500 TCID_50_ and simultaneously treated with 1 mg/mL of glycyrrhizin. Supernatants were collected at seven different time points (0 h, 4 h, 8 h, 24 h, 28 h, 32 h and 48 h) post-infection. Viral RNA was purified from the supernatants with the “High Pure Viral RNA Kit” (Roche Diagnostics, Basel, Switzerland), and the genomic SARS-CoV-2 RNA was quantified via RT-qPCR. Therefore, primer targeting the viral M or N gene were used [7]. M and N gene copy numbers were assessed using a 1:10 plasmid dilution series as reference (details and sequence information available upon request).

### 2.8. Determination of Main Protease Inhibition

The inhibition of SARS-CoV-2 M^pro^ by glycyrrhizin was measured using the “Fluorogenic 3CL Protease, MBP-tagged (SARS-CoV-2) Assay Kit” (BPS Bioscience, San Diego, CA, USA). Briefly, 90 ng of recombinant SARS-CoV-2 M^pro^ were incubated with two different concentrations of glycyrrhizin (30 µM and 2000 µM, dissolved in water) in a 96-well microtiter plate at room temperature for 30 min. As control, the protease inhibitor GC376 was used. The enzyme activity was measured using Mithras LB 943 (Berthold Technologies, Oak Ridge, TN, USA) at 360 nm excitation and 460 nm emission after overnight incubation of the inhibitor-M^pro^ mixtures with the added substrate (Dabcyl-KTSAVLQ↓SGFRKM-E(Edans)-NH2) at room temperature. Data were analyzed using GraphPad Prism 8.0.1 (Graph Pad Software, San Diego, CA, United States). Statistical analysis of inhibition of the protease activity was performed with non-parametric ANOVA (Kruskal–Wallis) and post hoc Dunn’s multiple-comparisons test. Comparisons were considered significant at * *p* < 0.05, ** *p* < 0.01 and *** *p* < 0.001.

## 3. Results

### 3.1. Antiviral Activity of Licorice Root Extract

**Figure 1:**
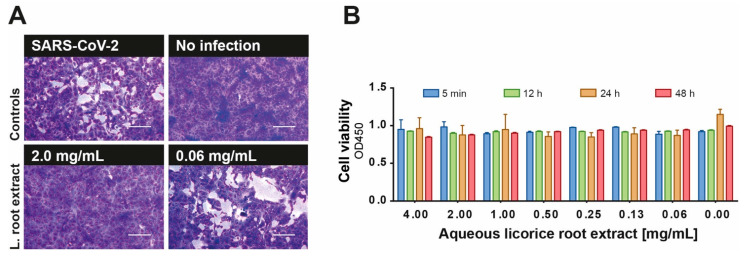
Potent inhibition of SARS-CoV-2 replication by the licorice root extract. (**A**) Decreasing aqueous licorice root extract concentrations (0.004–4 mg/mL) were pre-incubated with 100 TCID_50_/mL SARS-CoV-2 for 1 h at 37 °C and applied to a confluent layer of Vero E6 cells. After 48 h of incubation, cell cultures were stained with crystal violet and analyzed for plaque formation. Representative pictures of protected and infected cell cultures at the indicated concentrations are shown. Bars represent 200 µm. (**B**) Cytotoxicity of aqueous licorice root extract towards Vero E6 cells was tested using “Orangu cell counting solution”. Different concentrations of the extract were incubated with a confluent layer of Vero E6 cells and the cell viability was evaluated at four different time points (5 min, 12 h, 24 h, 48 h). All experiments were performed in biological replicates. Error bars represent the standard error of the mean (SEM).

Licorice root extract is of great importance in traditional medicine and was shown to be effective against coronaviruses and other viruses [3,4]. In the present study, we investigated the antiviral efficacy of licorice root as a part of the Glycyrrhiza glabra plant against SARS-CoV-2. Initially, we investigated the antiviral activity of an aqueous licorice root extract against SARS-CoV-2 in vitro. Various concentrations of the extract (0.004–4 mg/mL) were pre-incubated with a viral load of 100 TCID_50_ of SARS-CoV-2 for 1 h and subsequently added to confluent Vero E6 cells. Plaque formation was visualized after 48 h of infection using crystal violet. Aqueous licorice root extract showed antiviral effects even at a subtoxic concentration of 2 mg/mL (Figure 1A,B). This concentration is lower than the normal consuming dilution, e.g., in tea (12.5 mg/mL). Although licorice root tea may represent a good candidate for complementary use, the identification and characterization of the active compound is of great importance for a potential clinical application.

### 3.2. Effective Inhibition of SARS-Cov-2 Replication by Glycyrrhizin

**Figure 2:**
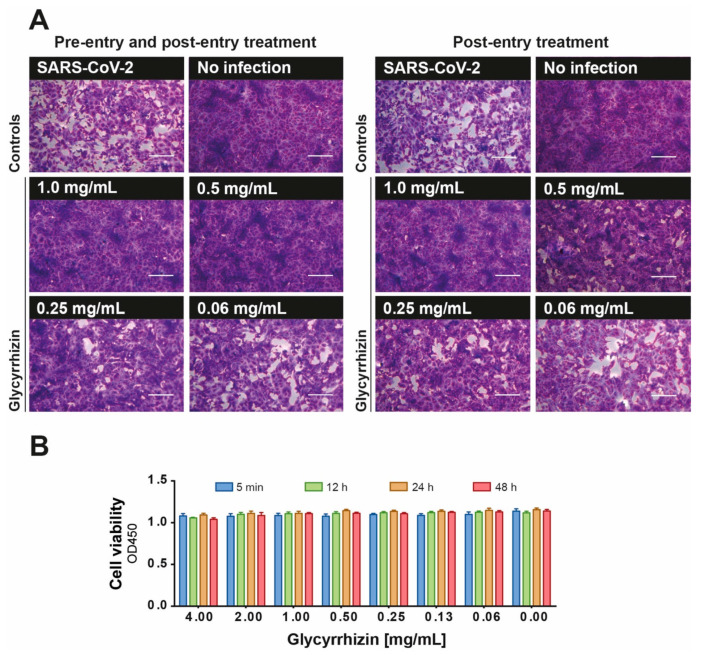
Antiviral efficacy of glycyrrhizin against SARS-CoV-2. The antiviral efficacy of glycyrrhizin against SARS-CoV-2 was investigated under combined pre- and post-entry or post-entry conditions. (**A**) Descending concentrations of glycyrrhizin (0.002–4 mg/mL) were pre-incubated with 100 TCID_50_ SARS-CoV-2 for 1 h at 37 °C and subsequently added to confluent Vero E6 cells in 96-well microtiter plates for 48 h (combined pre- and post-entry conditions). In a second approach, Vero E6 cells were inoculated with 100 TCID_50_ SARS-CoV-2 for 4 h before the glycyrrhizin-containing inoculation medium with various glycyrrhizin concentrations (0.002–4 mg/mL end-concentration) was added (post-entry conditions). Plaque formation was evaluated after 48 h post infection (p.i.). Bars represent 200 µm. (**B**) Cytotoxicity of glycyrrhizin used at the indicated concentrations towards Vero E6 cells was tested using “Orangu cell counting solution”. Different concentrations of glycyrrhizin were incubated with a confluent layer of Vero E6 cells and the cell viability was evaluated at four different time points (5 min, 12 h, 24 h, 48 h). All experiments were performed in biological replicates. Error bars represent the standard error of the mean (SEM).

Glycyrrhizin, the main ingredient of the licorice root, was shown to exhibit antiviral activity against viruses such as herpes simplex virus, human immunodeficient virus and other coronaviruses [3,4]. Therefore, we hypothesized that aqueous licorice root extract may inhibit SARS-CoV-2 replication due to its high content of glycyrrhizin. For this reason, we investigated its antiviral activity of glycyrrhizin against SARS-CoV-2 in vitro under pre- and post-entry conditions. Descending concentrations of glycyrrhizin (0.002–4 mg/mL) were pre-incubated with 100 TCID_50_ SARS-CoV-2 for 1 h at 37 °C and the mixtures were subsequently incubated on confluent Vero E6 cells for 48 h (combined pre- and post-entry conditions). Additionally, Vero E6 cells were inoculated with 100 TCID_50_ SARS-CoV-2 for 4 h before the glycyrrhizin-containing inoculation medium with various glycyrrhizin concentrations (0.002–4 mg/mL end-concentration) was added (post-entry conditions). Plaque formation was evaluated after 48 h post infection (p.i.). SARS-CoV-2 replication was completely blocked by glycyrrhizin at a concentration of 0.5 mg/mL (combined pre- and post-entry conditions) or 1 mg/mL (post-entry conditions) (Figure 2A).

In order to exclude the possibility of toxic-associated effects of glycyrrhizin to Vero E6 cells, we analyzed the impact of various concentrations of glycyrrhizin on the cell viability. No cytotoxic effect could be observed even at a concentration of 4 mg/mL (Figure 2B).

### 3.3. Quantitative Analysis of the Antiviral Activity of Glycyrrhizin Against SARS-Cov-2

**Figure 3:**
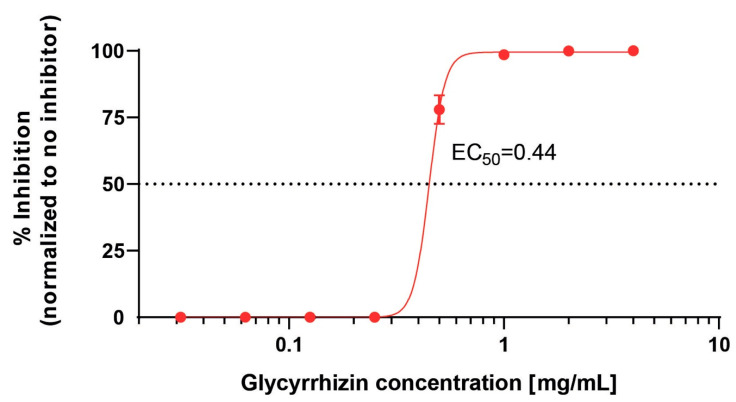
Dose-dependent activity of glycyrrhizin against SARS-CoV-2. Vero E6 cells were infected with 10,000 TCID_50_ and treated with serial dilutions of glycyrrhizin (0.004−4 mg/mL), respectively. After 48 h of infection, cell culture supernatants were harvested and the viral loads were determined using endpoint dilution. The experiment was performed in technical triplicates. The EC_50_ value was calculated using GraphPad Prism. Error bars display the standard error of the mean (SEM).

**Figure 4:**
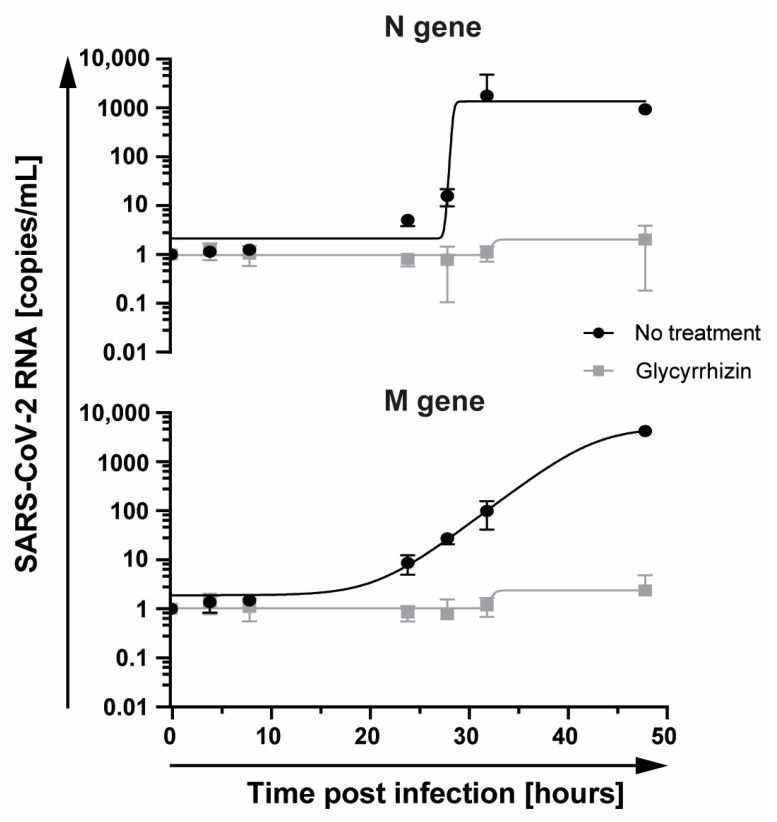
Glycyrrhizin treatment reduces SARS-CoV-2 RNA levels in cell culture supernatant. Vero E6 cells were infected with 500 TCID_50_ SARS CoV-2 and treated with glycyrrhizin at 1 mg/mL. Total RNA was extracted from the supernatants harvested at 0, 4, 8, 24, 28, 32 and 48 h post infection. SARS-CoV-2 RNA was quantified by determining the amounts of the M and N gene using RT-qPCR. All experiments were performed in biological replicates. Error bars displays the standard error of the mean (SEM).

Next, we determined the half-maximal effective concentration (EC_50_) of glycyrrhizin against SARS-CoV-2 and investigated the impact of glycyrrhizin on SARS-CoV-2 replication on RNA level. Therefore, confluent Vero E6 cells were infected with 10,000 TCID_50_ SARS-CoV-2 and treated with various concentrations of glycyrrhizin ranging from 0.0625 to 4 mg/mL. After 48 h, the cell culture supernatants were titrated and the EC_50_ value was determined. The EC_50_ was calculated with 0.44 mg/mL, uncovering glycyrrhizin as a potent compound effective against SARS-CoV-2 (Figure 3). The initial finding was supported by quantifying the SARS-CoV-2 RNA from the supernatants of SARS-CoV-2 infected cells treated with glycyrrhizin. Vero E6 cells were infected with 1 × 10^4^ TCID_50_ SARS-CoV-2 and treated with 1 mg/mL glycyrrhizin. SARS-CoV-2 RNA levels were quantified at 0, 4, 8, 24, 28, 32 and 48 h post infection. Untreated Vero E6 cells served as control. Glycyrrhizin treatment significantly reduced SARS-CoV-2 RNA levels in cell culture supernatants, indicating a potent antiviral activity of glycyrrhizin against SARS-CoV-2 (Figure 4). Taken together, we demonstrated that glycyrrhizin exhibited a high antiviral activity against SARS-CoV-2 and completely inhibited the viral replication at subtoxic concentrations.

### 3.4. Inhibition of the Viral Main Protease by Glycyrrhizin

**Figure 5:**
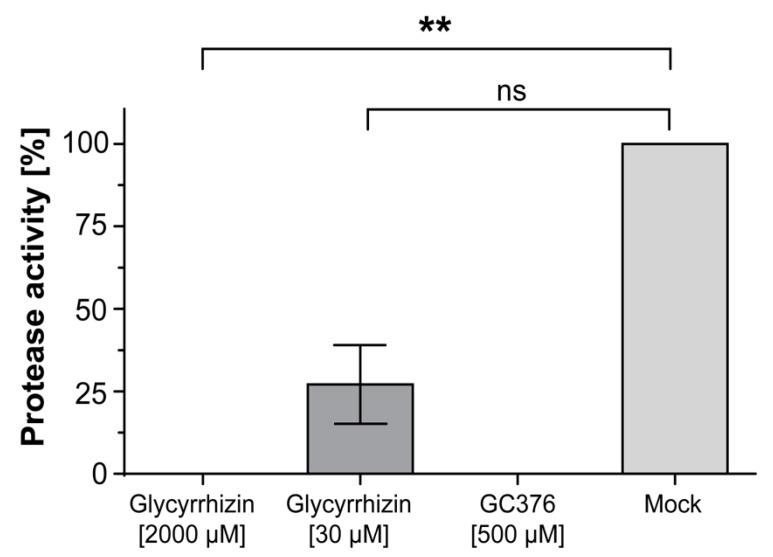
Glycyrrhizin potently inhibits the viral main protease (M^pro^). The inhibition of SARS-CoV-2 M^pro^ by glycyrrhizin was measured using the “Fluorogenic 3CL Protease, MBP-tagged (SARS-CoV-2) Assay Kit”. Different glycyrrhizin concentrations (30 µM and 2000 µM) and the complete protease inhibitor GC376 (100 µM) were dissolved with 90 ng M^pro^ in 30 µL 0.5 M DTT Buffer and incubated for 30 min at room temperature. Subsequently, the 3CL Protease (SARS-CoV-1/SARS-CoV-2) substrate was added and the protease activity was measured after overnight incubation at a wavelength of 360 nm/460nm (*exc/em*). The experiment was performed in triplicates. Measurement was performed using Mithras LB 943 after 12 h incubation. Statistical analysis was undertaken with a non-parametric ANOVA test. Comparisons were considered significant at ** *p* < 0.01 and n.s. = no significance. All experiments were performed in biological replicates. Error bars represent the standard derivation of the mean (SD).

Next, we investigated the underlying mechanism of how glycyrrhizin may interfere with the virus replication. Recently, protease inhibitory activity of glycyrrhizin was predicted using in silico simulations [8]. The SARS-CoV-2 main protease M^pro^, also known as the 3CL protease, plays a vital role in processing the viral polyproteins that are translated from SARS-CoV-2 RNA. This process is essential for the virus replication. Glycyrrhizin was suggested as a possible inhibitor of the viral main protease M^pro^, but this hypothesis has not been experimentally proven, yet [9]. Thus, we investigated whether glycyrrhizin may inhibit the proteolytic activity of the SARS-CoV-2 M^pro^ by using a 3CL protease activity kit [10]. Different glycyrrhizin concentrations (30 µM and 2000 µM) were dissolved in 0.5 M DTT Buffer containing Mpro at a concentration of 3 ng/µL and incubated for 30 min at room temperature. As control, the protease inhibitor GC376 was used at 100 µM. Subsequently, the 3CL Protease (SARS-CoV-1/SARS-CoV-2) substrate was added and the mixture was incubated overnight. Protease activity was measured by determining the enzymatic reaction of the substrate at OD 360nm/460nm (*exc/em*). Glycyrrhizin completely inhibited M^pro^ activity at a concentration of 2000 µM (1.6 mg/mL) and reduced its activity by 70.3% at a concentration of 30 µM (0.024 mg/mL) (Figure 5). For the first time, we could demonstrate that glycyrrhizin inhibits the SARS-CoV-2 main protease (3CL protease), thereby potently blocking the viral replication.

## 4. Discussion

The newly emerged coronavirus SARS-CoV-2 is continuing to spread around the world thereby causing major public health issues. Remdesivir is currently the only medication approved by the FDA to treat COVID-19. Corticosteroids such as dexamethasone are used to treat the sickest patients with COVID-19 suffering from a hyperinflammatory immune response [11]. Despite some recently approved vaccines in the last months, there is still a great medical need for novel, well tolerated and broadly accessible interventions. Herbal medicine revealed to be a promising option for the treatment of various viral infections. In the present study, we investigated the antiviral efficacy of licorice root and its main ingredient glycyrrhizin against SARS-CoV-2. We demonstrated that glycyrrhizin potently inhibits SARS-CoV-2 replication in cell culture at subtoxic concentrations and identified the underlying mechanism.

Traditional medicine was shown to be of great importance in the development of modern medication. The primary active compound of the licorice root, glycyrrhizin, was evaluated as a liver protective in the treatment of chronic hepatitis B and C patients [12,13]. Of note, glycyrrhizin was clinically evaluated in the context of pharmacokinetic analyses and described to be a safe and well-tolerated compound [14]. The pharmacological effects include antioxidative and anti-inflammatory, corticosteroid-like activities [15]. The side effects of glycyrrhizin, such as hypertension, fatal arrhythmias and renal failure after several weeks of consumption, are well known and are therefore predictable and controllable [16]. Clinical trials with Stronger Neo-Minophagen C (SNMC), which is an intravenous pharmaceutical with glycyrrhizin as the primary active compound, showed liver protective results in hepatitis B patients [12]. In prior studies, different mechanisms causing the beneficial effect of glycyrrhizin were discussed. The anti-inflammatory and mineralocorticoid potency could be explained by the inhibition of 11-beta-hydroxysteroid dehydrogenase (11βHSDH) leading to a higher cortisol levels [17]. The potent antiviral activity as well as anti-inflammatory properties highlight glycyrrhizin as an excellent candidate for further clinical investigations in COVID-19 treatment.

The pharmacokinetic in rats showed that enterohepatic metabolism and biliary excretion play a major role in the drug clearance, and higher bioavailability of glycyrrhizin is given after intravenous or intraperitoneal than oral application [18]. After oral administration of 1500 mg of glycyrrhizin acid in healthy humans, the mean plasma concentration after 24 h was 4 mg/mL, which is more than four times higher than the calculated EC_50_ value determined for the inhibition of SARS-CoV-2 replication in our study [19]. These findings demonstrate that antiviral active levels of glycyrrhizin are reachable and well tolerated in humans. The drug’s half-time (*t_1/2_*) of glycyrrhizin in healthy males was described as 3.5 h^18^. In contrast, in human patients with chronic hepatitis C glycyrrhizin’s half-live after intravenous application was around 9 h [13]. In this study group, pharmacokinetics was linear up to 200 mg and steady-state was reached after 12 doses of 200 mg glycyrrhizin per day^19^. However, the glycyrrhizin dose required to reach a therapeutically effective concentration in the respiratory tract needs to be determined in subsequent studies. A case report described compassionate use of glycyrrhizin among other potential antivirals for the treatment of COVID-19 [20]. The patient received 150 mg glycyrrhizin 3 times per day for 8 days. The patient’s condition started improving after 12 h of treatment [20]. Although the patient recovered from disease, further controlled studies are needed to prove the therapeutic effects of glycyrrhizin in COVID-19.

Glycyrrhizin was discussed in in silico simulations as a potential protease inhibitor [8,9]. Beside the viral main protease (M^pro^), the human transmembrane serine protease (TMPRSS2) is another discussed target of glycyrrhizin. TMPRSS2 was shown to cleave the SARS-CoV-2 spike protein thereby facilitating the entry of the virus into the host cell [21]. However, since there was only a slight difference in antiviral activity of glycyrrhizin between pre- and post-entry conditions, and only a minor affinity was simulated for the interaction between glycyrrhizin and TMPRSS2 in former in silico studies [21], we concluded that glycyrrhizin blocks SARS-CoV-2 replication mainly via a mechanism different from inhibiting TMPRSS2. Thus, we focused on the SARS-CoV-2 main protease (M^pro^) as a potential target for glycyrrhizin [22]. M^pro^ is essential for processing the viral polyproteins that are translated from the viral RNA and, thus, for virus replication [22]. Therefore, the inhibition of the SARS-CoV-2 main protease would inhibit the viral replication. Our experiments uncovered the inhibitory activity of glycyrrhizin against M^pro^ and confirm former data from different in silico simulations [8,22]. An inhibitory activity of glycyrrhizin towards TMPRSS2 or other enzymes cannot be completely excluded. However, since there was only a slight difference in the concentration of glycyrrhizin required for complete inhibition of viral replication under combined pre- and post-entry (0.5 mg/mL) or post-entry (1 mg/mL) conditions, we concluded that glycyrrhizin blocks SARS-CoV-2 replication mainly by inhibiting the main protease M^pro^.

Taken together, we demonstrated that glycyrrhizin, the primary active ingredient of the licorice root, potently blocks SARS-CoV-2 replication by inhibiting the viral main protease. Our experiments highlight glycyrrhizin as a potential antiviral compound that should be further investigated for the treatment of COVID-19.

## Data Availability

The data presented in this study are available on request from the corresponding author.
